# Prevalence and Correlates of Cardiovascular Calcification and Its Prognostic Effects Among Patients With Chronic Kidney Disease: Results From the C-STRIDE Study

**DOI:** 10.3389/fpubh.2021.762370

**Published:** 2022-01-06

**Authors:** Lan Wang, Hong Cheng, Xinrong Zou, Jun Yuan, Wenjing Wu, Siping Han, Jinwei Wang, Luxia Zhang, Kevin He, Ming-Hui Zhao, Xiaoqin Wang

**Affiliations:** ^1^The First Clinical College, Hubei University of Chinese Medicine, Wuhan, China; ^2^Institute of Chinese Medicine Nephrology, Hubei Provincial Hospital of TCM, Hubei Province Academy of Traditional Chinese Medicine, Wuhan, China; ^3^Renal Division, Department of Medicine, Peking University First Hospital, Beijing, China; ^4^Institute of Nephrology, Peking University, Beijing, China; ^5^Key Laboratory of Renal Disease, National Health Commission of China, Beijing, China; ^6^Key Laboratory of Chronic Kidney Disease Prevention and Treatment (Peking University), Ministry of Education, Beijing, China; ^7^Research Units of Diagnosis and Treatment of Immune-Mediated Kidney Diseases, Chinese Academy of Medical Sciences, Beijing, China; ^8^National Institute of Health Data Science at Peking University, Beijing, China; ^9^Department of Biostatistics, School of Public Health, University of Michigan, Ann Arbor, MI, United States; ^10^Peking-Tsinghua Center for Life Sciences, Beijing, China

**Keywords:** cardiovascular calcification, chronic kidney disease, abdominal aortic calcification, cardiac valve calcification, C-STRIDE

## Abstract

**Background and Aims:** The purpose of this study was to identify the characteristics and risk factors for cardiovascular calcification, and its relationship to prognosis, in patients with chronic kidney disease (CKD) stages 1–4.

**Methods:** Cardiovascular calcification was evaluated at baseline by lateral abdominal radiography to detect abdominal aortic calcifications (AAC), and by echocardiogram to detect cardiac valvular calcifications (CVC), respectively. Demographic and laboratory data were collected and analyzed. Univariate and multivariable logistic regression model was used to explore the factors associated with the indicators of cardiovascular calcification, while Cox proportional hazards regression was used to examine the association between AAC/CVC and incidence of cardiovascular events and all-cause mortality.

**Results:** A subgroup of 2,235 patients with measurement of AAC in the C-STRIDE study and a subgroup of 2,756 patients with CVC were included in the analysis. AAC was present in 206 patients (9.22%) and CVC was present in 163 patients (5.91%). Age, gender, history of cardiovascular diseases, smoking, hypertension, diabetes, levels of hemoglobin, low-density lipoprotein cholesterol, and uric acid were associated with prevalence of AAC, while only age, history of cardiovascular diseases, levels of serum albumin and low-density lipoprotein cholesterol were associated with prevalence of CVC (all *p* < 0.05).Survival analyses showed that cardiovascular events and all-cause mortality were significantly greater in patients with AACor with CVC (all p-values for log-rank tests <0.05). After adjustment for age, sex and estimated glomerular filtration rate (eGFR), AAC was associated with increased risk of all-cause mortality (hazard ratio = 1.67[95% confidence interval: 0.99, 2.79]), while CVC associated with that of cardiovascular events only among patients with comparatively normal eGFR (≥45 ml/min/1.73m^2^) (hazard ratio = 1.99 [0.98, 4.03]).

**Conclusion:** Demographic and traditional cardiovascular risk factors were associated with cardiovascular calcification, especially AAC. AAC may be associated with risk of death for patients CKD of any severity, while CVC as a possible risk factor for cardiovascular disease only among those with mild to moderate CKD. Assessments of vascular calcification are need to be advanced to patients in the early and middle stages of chronic kidney disease and to initiate appropriate preventive measures earlier.

## Introduction

Chronic kidney disease (CKD) has become a global public health problem, with a prevalence among the general population of more than 10% in both developed and developing countries (1). Cardiovascular disease (CVD) is a serious complication in CKD patients that is the leading cause of CKD associated mortality (2). Our previous study reported that the overall prevalence of CVD among 3,168 participants was 9.8% at enrollment and also showed that abdominal aortic calcification (AAC) was independently associated with CVD (3). It is widely acknowledged that cardiovascular calcification is closely associated with morbidity and mortality in patients with CVD. Many factors contribute to the pathogenesis of vascular calcifications (VC) (4). Of note, VC is common in CKD patients, and reduced renal function is known to facilitate VC progression (5). Given that VC has deleterious effects on clinical outcomes, the Kidney Disease Improving Global Outcome (KDIGO) experts suggest that vascular calcification should be considered as the highest cardiovascular risk factor in patients with CKD stages 3 to 5D (6). Therefore, exploring the risk factors associated with cardiovascular calcifications in CKD and their relationship with CVD prognosis is of great value for the prevention and treatment for CKD combined with CVD.

In clinical practice, there are multiple ways to evaluate calcification in the cardiovascular system, including lateral abdominal radiograph, echocardiogram, arterial pulse wave velocity, carotid intima-media thickness, electron-beam CT (EBCT), and multislice spiral computed tomography (MSCT) (7). Among them, EBCT and MSCT are regarded as the gold standard for VC diagnosis. However, due to their high cost and potential for radioactive injury, they are not considered to be routine methods. Hence, KDIGO guidelines suggest the use of lateral abdominal radiograph to detect aortic calcifications (2C) and echocardiogram to detect valvular calcifications (2C) (6).

To date, most studies have focused on AAC and cardiac valve calcification (CVC) found in dialysis patients. However, there are sparse data on AAC and CVC in non-dialysis CKD patients. We hypothesized that there is a close association between vascular calcification and the prognosis of non-dialysis CKD patients. To test this hypothesis, a prospective follow-up based on C-STRIDE study was conducted to identify the characteristics and risk factors associated with cardiovascular calcification, and its relationship to prognosis, in patients with CKD stages 1–4.

## Methods

### Participants

The C-STRIDE is an ongoing multicenter prospective study lead by the Peking University First Hospital that includes 39 clinical centers located in 28 cities in 22 provinces of China. The study design and methods have been published (8). This study was approved by the ethics committee of Peking University First Hospital. It strictly adhered to the Helsinki declaration, and all participants signed written informed consent.

Patient enrollment was carried out between November 2011 and March 2016. Altogether, 3,459 Chinese patients were enrolled with CKD stages 1–4 aged from 18 to 74 years old. Among them, a subgroup of 2,235 patients received lateral abdominal radiograph and a second subgroup of 2,756 patients underwent echocardiogram.

### Quality Control

Of note, there was unified operation manual in 39 clinical centers. Investigators from different centers received training by Peking University First Hospital.

### Data Collection

Covariates were selected based on prior clinical knowledge as potential confounders in the study. Demographic and clinical information on age, gender, health history (hypertension, diabetes, and cardiovascular disease), medication (phosphorus binder, active vitamin D preparations), smoking, body mass index (BMI) and resting blood pressure were recorded at entry. Echocardiogram, lateral abdominal radiograph and 24-h urine protein were performed with standardized procedures across all clinical centers. All blood measurements, including serum creatinine (SCr), uric acid, calcium, phosphorus, fasting glucose, triglyceride (TG), total cholesterol (TC), high density lipoprotein cholesterol (HDL-C), low density lipoprotein cholesterol (LDL-C), hemoglobin (Hb), and intact parathyroidhormone (iPTH) were performed in the central laboratory of Peking University First Hospital to avoid testing variations among different laboratories.

The KDIGO classification was used to determine the CKD stages (9).

The estimated glomerular filtration rate (eGFR) was calculated with CKD-EPI equation using SCr measured by the Roche enzymatic method (10). The eGFR of glomerulonephritis patients was defined to be ≥15 ml/min/1.73 m^2^, and for diabetic nephropathy patients, 15 ml/min/1.73 m^2^ ≤ eGFR <60 ml/min/1.73 m^2^ or eGFR≥ 60 ml/min/1.73 m^2^ with “nephrotic range” proteinuria including 24-h urinary protein ≥3.5 g or urinary albumin creatinine ratio (UACR) ≥2 000 mg/g.

### Detection of AAC and CVC

Lateral abdomen radiograph and echocardiogram were performed by experienced radiologists who were blinded to other patient data. A lateral plain radiograph of the abdomen was obtained and the aorta was identified as the tubular structure coursing in front of the anterior surface of the spine. AAC was defined as the presence of a longitudinal linear or strip-shaped high-density shadow at the level of the first to fourth lumbar vertebrae (11, 12). CVC was defined as the presence of bright echoes of more than 1 mm thickness on the aortic or mitral valves (13).

### Outcomes

Cardiovascular events included non-fatal myocardial infarction, unstable angina, cerebrovascular events (intraparenchymal hemorrhage, subarachnoid hemorrhage, cerebral infarction), hospitalization for congestive heart failure, serious cardiac arrhythmia (resuscitated cardiac arrest, ventricular fibrillation, sustained ventricular tachycardia, paroxysmal ventricular tachycardia, an initial episode of atrial fibrillation or flutter, severe bradycardia or heart block) and peripheral arterial diseases. The events were investigated by trained staff every 6 months through phone calls or routine clinical visits and confirmed by medical records. The patients who cannot be contacted for more than half a year were considered loss of follow-up, with the date of last follow-up used for censoring. The rates for loss of follow-up were 3.36% for the AAC dataset and 3.34% for the CVC dataset, respectively. An independent committee consisting of specialist physicians in Peking University First Hospital adjudicated the outcomes. The follow-up for the adverse cardiovascular events and all-cause mortality was through December 31, 2017. If several CVD events occurred, the first event was used as the index event. The CVD events were censored at death, loss of follow-up or December 31, 2017, while death was censored at loss of follow-up or the above administrative end of follow-up.

### Statistical Analysis

Baseline values are presented as mean ± standard deviation (SD) or medians and interquartile ranges for continuous variables, and as numbers and percentages for categorical data. Baseline characteristics were compared between groups using analysis of variance (ANOVA), Kruskal-Wallis rank sum test or chi-square tests, as appropriate. Logistic regression analysis was used to identify factors associated with AAC or CVC. Both univariate and multivariable regressions were conducted and all variables regardless of statistical significance in univariate analysis were included in multivariable analysis. Before entering the regression models, missing values were filled with mean or median for continuous variables or with a separate category for categorical variables and values of variables with skewed distribution were natural logarithm transformed. Survival analysis was conducted and the difference of incident rates between groups was compared using log-rank test. Kaplan-Meier curve was also depicted. The association between AAC/CVC and outcomes was examined by using the Cox proportional hazards regression models. A series of models were conducted with univariate analysis in model 1, adjustment of age and sex in model 2 and additional adjustment of eGFR in model 3. In order to test the interaction between eGFR and the main exposure variables on the association, we included interaction term between eGFR (as a continuous variable) and AAC/CVC status in the regression model in addition to the two explanatory variables themselves. The assumption of proportional hazards was tested by using Schoenfeld residuals, with no violations detected for the independent variables. The *p* < 0.05 was considered statistically significant. SAS software (version 9.4, SAS Institute Inc, Cary, NC) was used for statistical analysis.

## Results

### Baseline Characteristics by Presence of AAC or CVC

**Figure 1:**
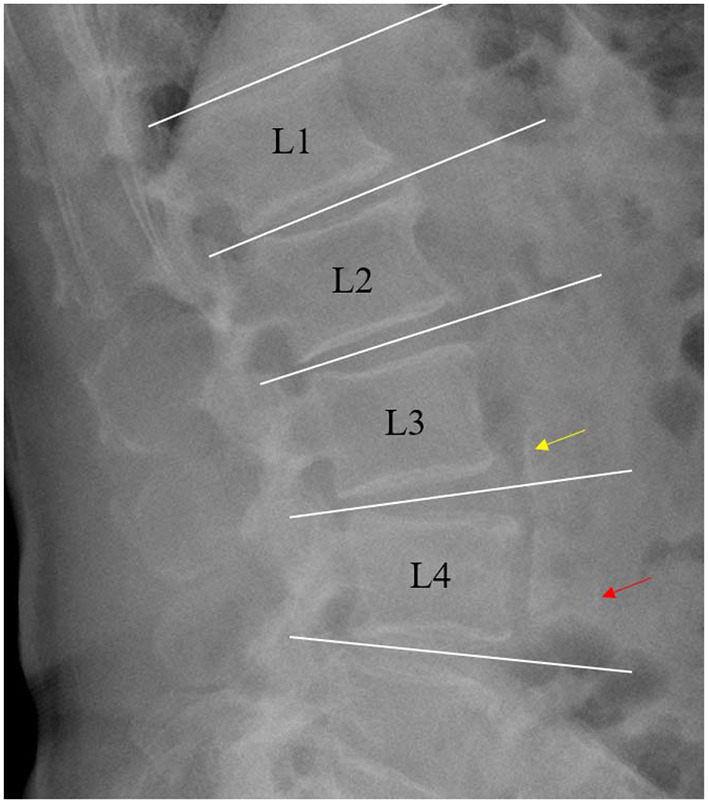
Example of lateral abdomen radiograph showing calcification of the anterior (red arrow) and posterior wall (yellow arrow) of the abdominal aorta.

Altogether, 2,235 patients with CKD stages 1–4 and with measurement of lateral abdomen radiograph were included in the present study (Figure 1). The proportion of AAC in these patients was 9.22%.Patients with AAC were significant older than those without (*p* < 0.001). Prevalence of diabetes and hypertension was significantly greater in patients with than without AAC (*p* < 0.001). Patients with AAC had a higher prevalence of CVD than those without (27.18 vs. 9.25%, *p* < 0.001). Hemoglobin levels were significantly lower in patients with AAC compared to those without, whereas serum uric acid and iPTH levels were significantly higher. BMI, smoking and eGFR were also different with and without AAC. In addition, significant differences were noted in the use of calcium-free phosphorus binder and active vitamin D preparations between patients with and without AAC (10.68 vs. 17.00%, *P* = 0.02; 7.77 vs. 12.96%, *P* = 0.03). Of 2,136 patients receiving echocardiogram in the current subgroup, 126 patients had CVC. The prevalence of CVC was significantly higher in patients with AAC than without AAC (15.18 vs. 4.99%, *p* < 0.001) (Table 1A).

**Table 1:** **(A)** Characteristics of patients with non-dialysis CKD Stages 1–4 with and without AAC at baseline. **(B)** Characteristics of patients with non-dialysis CKD Stages1 to 4 with and without CVC at baseline.

**Variable**	**Total (*n =* 2,235)**	**Abdominal aortic calcification**		**Missing value**	***P***
		**Yes (*n =* 206)**	**No (*n =* 2,029)**		
**(A)**					
Age (years)	48.34 ± 13.67	60.64 ± 10.30	47.09 ± 13.35	0	<0.001
Male gender (%)	1,304 (58.34%)	115 (55.83%)	1189 (58.60%)	0	0.44
BMI (kg/m^2^)	24.33 ± 3.59	24.83 ± 3.49	24.27 ± 3.60	51	0.04
History of CVD	243 (10.91%)	56 (27.18%)	187 (9.25%)	8	<0.001
Tobacco use (%)	847 (38.41%)	92 (46.23%)	755 (37.64%)	30	0.008
Hemoglobin (g/L)	127.52 ± 23.22	117.81 ± 23.32	128.50 ± 22.99	27	<0.001
Serum albumin (g/L)	38.31 ± 7.50	38.44 ± 6.94	38.29 ± 7.56	85	0.80
Urinary protein g/24 h	0.93 (0.34, 2.25)	0.98 (0.43, 2.30)	0.92 (0.34, 2.23)	113	0.27
Diabetes (%)	470 (21.03%)	99 (48.06%)	371 (18.28%)	0	<0.001
Hypertension (%)	1,574 (77.12%)	171 (91.44%)	1,403 (75.67%)	194	<0.001
Cholesterol (mmol/L)	4.71 (3.90, 5.80)	4.74 (3.89, 5.82)	4.71 (3.90,5.80)	96	0.90
Triglyceride (mmol/L)	1.81 (1.26, 2.60)	1.85 (1.33, 2.42)	1.80 (1.26, 2.62)	107	0.98
LDL-C(mmol/L)	2.57 (2.05, 3.23)	2.7 (2.13, 3.23)	2.56 (2.04, 3.23)	136	0.16
Serum calcium (mmol/L)	2.22 ± 0.21	2.22 ± 0.22	2.22 ± 0.21	98	0.86
Serum phosphorus (mmol/L)	1.19 (1.05, 1.34)	1.18 (1.05, 1.39)	1.19 (1.05, 1.33)	99	0.75
IPTH(pg/ml)	44.48 (28.16,71.10)	54.24 (33.29,82.00)	43.39 (27.81,69.79)	356	<0.001
**Medication**					
Calcium-free Phosphorus binder	367 (16.42%)	22 (10.68%)	345 (17.00%)	0	0.02
Active vitamin D	279 (12.48%)	16 (7.77%)	263 (12.96%)	0	0.03
Uric acid (umol/L)	401.74 ± 122.73	428.62 ± 110.82	399.00 ± 123.57	42	0.001
eGFR (ml/min/1.73 m^2^)	54.30 ± 34.88	41.51 ± 25.40	55.60 ± 35.45	0	<0.001
eGFR staging					
eGFR≥90 (*n*%)	373 (16.69%)	13 (6.31%)	360 (17.74%)		
60 ≤ eGFR <90 (*n*%)	385 (17.23%)	18 (8.74%)	367 (18.09%)		
45 ≤ eGFR <60 (*n*%)	342 (15.3%)	35 (16.99%)	307 (15.13%)		
30 ≤ eGFR <45 (*n*%)	506 (22.64%)	65 (31.55%)	441 (21.73%)		
15 ≤ eGFR <30 (*n*%)	629 (28.14%)	75 (36.41%)	554 (27.3%)		
Cardiac valve calcification (*n*%)	126 (5.90%)	29 (15.18%)	97 (4.99%)	99	<0.001
**Variable**	**Total (*****n** **=*** **2,756)**	**Cardiac valve calcification**		**Missing value**	***P***
		**Yes (*n* = 163)**	**No (*n* = 2,593)**		
**(B)**					
Age (years)	48.73 ± 13.73	63.60 ± 8.30	47.80 ± 13.46	0	<0.001
Male gender (%)	1,613 (58.53%)	93 (57.06%)	1,520 (58.62%)	0	0.69
BMI (kg/m^2^)	24.38 ± 3.61	25.03 ± 3.49	24.34 ± 3.61	197	0.02
History of CVD	339 (12.35%)	51 (31.29%)	268 (10.38%)	10	<0.001
Tobacco use (%)	1,023 (37.85%)	61 (39.61%)	962 (37.74%)	53	0.64
Hemoglobin (g/L)	127.36 ± 23.16	120.78 ± 20.38	127.79 ± 23.27	179	<0.001
Serum albumin (g/L)	38.81 ± 7.32	37.57 ± 7.59	38.89 ± 7.30	124	0.03
Urinary protein g/24 h	0.92 (0.32, 2.23)	0.80 (0.23, 2.42)	0.94 (0.33, 2.22)	293	0.19
Diabetes (%)	590 (21.41%)	64 (39.26%)	526 (20.29%)	0	<0.001
Hypertension (%)	1,839 (77.30%)	127 (83.55%)	1,712 (76.87%)	377	0.06
Cholesterol (mmol/L)	4.72 (3.91, 5.80)	4.62 (3.56, 5.41)	4.74 (3.93, 5.82)	164	0.03
Triglyceride (mmol/L)	1.79 (1.26, 2.59)	1.75 (1.30, 2.61)	1.79 (1.25, 2.59)	164	0.95
LDL-C(mmol/L)	2.57 (2.05, 3.23)	2.41 (1.78, 2.97)	2.58 (2.07, 3.24)	205	0.002
**(B)**					
Serum calcium (mmol/L)	2.227 ± 0.203	2.22 ± 0.27	2.23 ± 0.20	131	0.47
Serum phosphorus (mmol/L)	1.18 (1.05, 1.33)	1.21 (1.03, 1.31)	1.18 (1.05, 1.33)	141	0.96
IPTH(pg/ml)	46.60 (29.60, 73.88)	48.25 (35.84, 73.33)	46.53 (29.14, 74.27)	493	0.19
**Medication**					
Calcium-free Phosphorus binder	485 (17.60%)	33 (20.25%)	452 (17.43%)	0	0.36
Active vitamin D	404 (14.66%)	32 (19.63%)	372 (14.35%)	0	0.06
Uric acid (umol/L)	404.46 ± 119.79	402.07 ± 128.30	404.61 ± 119.26	47	0.800
eGFR (ml/min/1.73m^2^)	52.43 ± 33.47	40.71 ± 22.13	53.17 ± 33.92	0	<0.001
**eGFR staging**					
eGFR≥90 (*n*%)	412 (14.95%)	4 (2.45%)	408 (15.73%)		
60 ≤ eGFR <90 (*n*%)	453 (16.44%)	19 (11.66%)	434 (16.74%)		
45 ≤ eGFR <60 (*n*%)	442 (16.04%)	35 (21.47%)	407 (15.70%)		
30 ≤ eGFR <45 (*n*%)	653 (23.69%)	51 (31.29%)	602 (23.22%)		
15 ≤ eGFR <30 (*n*%)	796 (28.88%)	54 (33.13%)	742 (28.62%)		

*BMI, body mass index; CVD, cardiovascular disease; LDL-C, low-density lipoprotein cholesterol; IPTH: intact parathyroid hormone; eGFR, estimated glomerular filtration rate*.

Three types of etiology (nephritis, diabetic kidney disease and other etiologies) were recorded at entry. In AAC subgroup, the proportion of diabetic kidney disease in these patients with AAC was 25.82%, compared to the proportion of nephritis (4.78%) and of other etiologies (12.29%), significant differences were noted (*p* < 0.001).

**Figure 2:**
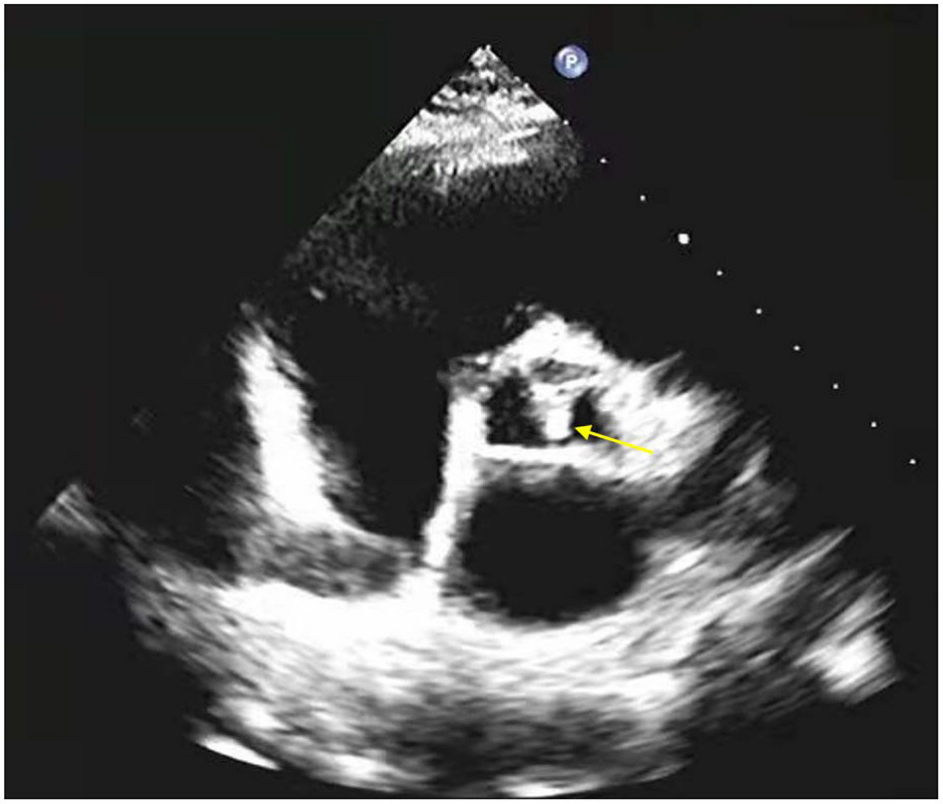
Example of echocardiogram showing calcification of the aortic valve (yellow arrow).

Altogether, 2,756 patients with CKD stages 1–4 and with measurement of echocardiogram were included in the present study (Figure 2). Among them, the proportion of patients with CVC was 5.91%. Patients with CVC were significant older than those without (*p* < 0.001). Prevalence of diabetes was significantly greater in patients with than without CVC (*p* < 0.001). Patients with CVC had a higher prevalence of CVD than those without (29.82 vs. 11.23%, *p* < 0.001). Hemoglobin and serum albumin levels were significantly lower in patients with CVC compared to those without. BMI and eGFR were also different with and without CVC. In addition, significant differences were noted in cholesterol and LDL-C between patients with and without CVC (*P* = 0.028; *P* = 0.002) (Table 1B).

In CVC subgroup, the proportion of diabetic kidney disease in these patients with CVC was 13.04%, compared to the proportion of nephritis (3.05%) and of other etiologies (9.37%), significant differences were noted between CVC patients with diabetic kidney disease and with nephritis (*p* < 0.001). But, lost statistical significance between CVC patients with diabetic kidney disease and other etiologies (*P* = 0.068).

### Associations Between Risk Factors and AAC or CVC

Results of univariate logistic regression were shown in Supplementary Tables 1, 2. In the multivariable analyses, older age, female, history of CVD, smoking, lower hemoglobin, hypertension, diabetes, higher LDL levels and higher serum uric acid levels were significantly associated with AAC in CKD non-dialysis patients (Table 2A). Regarding CVC, older age, history of CVD, lower albumin and decreasing LDL were found to be independently associated withthe abnormality in patients with CKD Stages 1–4 (Table 2B).

**Table 2:** **(A)** Multivariable logistic regression analysis for AAC in patients with CKD Stages 1–4. **(B)** Multivariable logistic regression analysis for CVC in patients with CKD Stages 1–4.

**Characteristic**	**OR**	**95%CI**	***P***
**(A)**			
Age, per 1 year increase	1.08	1.06–1.10	<0.001
Gender, male vs. female	0.63	0.39–1.01	0.05
BMI, per 1 kg/m^2^	1.01	0.96–1.06	0.838
History of CVD, yes vs. no	1.76	1.20–2.59	0.004
Tobacco use, yes vs. no	1.73	1.10–2.70	0.03
Hemoglobin, per 1 g/L increase	0.99	0.98–1.00	0.003
Serum albumin, per 1 g/L increase	1.03	1.00–1.07	0.08
24–h urine protein, per 1 natural log increase (g/24 h)	1.09	0.95–1.25	0.22
Hypertension, yes vs. no	1.90	1.08–3.32	0.03
Diabetes, yes vs. no	1.87	1.33–2.65	<0.001
Triglyceride, per 1 natural log increase (mmol/L)	1.01	0.73–1.40	0.95
Cholesterol, per 1 natural log increase (mmol/L)	0.72	0.43–1.20	0.21
LDL–C, per 1 natural log increase (mmol/L)	1.89	1.11–3.21	0.02
eGFR, per 1 ml/min/1.73m^2^ increase	1.003	0.995–1.010	0.50
Serum uric acid, per 1 umol/L increase	1.002	1.000–1.004	0.02
Serum calcium, per 1 mmol/L increase	0.61	0.21–1.77	0.36
Serum phosphorus, per 1 natural log increase (mmol/L)	0.98	0.43–2.24	0.97
IPTH, per 1 natural log increase (pg/ml)	0.94	0.72–1.23	0.65
Calcium–free phosphorus binder, yes vs. no	0.75	0.44–1.26	0.27
Active vitamin D, yes vs. no	0.60	0.33–1.10	0.10
**(B)**			
Age, per 1 year increase	1.125	1.10–1.15	<0.001
Gender, male vs. female	1.022	0.63–1.66	0.93
BMI, per 1 kg/m^2^	1.02	0.97–1.08	0.46
History of CVD, yes vs. no	1.56	1.05–2.33	0.03
Tobacco use, yes vs. no	1.10	0.69–1.76	0.69
Hemoglobin, per 1 g/L increase	1.00	0.99–1.01	0.80
Serum albumin, per 1 g/L increase	0.96	0.92–1.00	0.03
24–h urine protein, per 1 natural log increase (g/24 h)	1.01	0.87–1.16	0.95
Hypertension, yes vs. no	0.77	0.47–1.25	0.29
Diabetes, yes vs. no	1.00	0.68–1.46	0.98
Triglyceride, per 1 natural log increase (mmol/L)	1.30	0.92–1.85	0.14
Cholesterol, per 1 natural log increase (mmol/L)	1.21	0.70–2.09	0.51
LDL–C, per 1 natural log increase (mmol/L)	0.42	0.24–0.73	0.002
eGFR, per 1 ml/min/1.73m^2^ increase	0.994	0.985–1.003	0.19
Serum uric acid, per 1 umol/L increase	0.998	0.997–1.000	0.08
Serum calcium, per 1 mmol/L increase	2.37	0.75–7.53	0.14
Serum phosphorus, per 1 natural log increase (mmol/L)	1.14	0.45–2.85	0.79
IPTH, per 1 natural log increase (pg/ml)	0.86	0.65–1.15	0.31
Calcium–free phosphorus binder, yes vs. no	0.97	0.59–1.58	0.89
Active vitamin D, yes vs. no	1.30	0.78–2.17	0.31

*BMI, body mass index; CVD, cardiovascular disease; LDL–C, low–density lipoprotein cholesterol; eGFR, estimated glomerular filtration rate, IPTH: intact parathyroid hormone*.

### Association of AAC or CVC With Cardiovascular Events or Death

**Figure 3:**
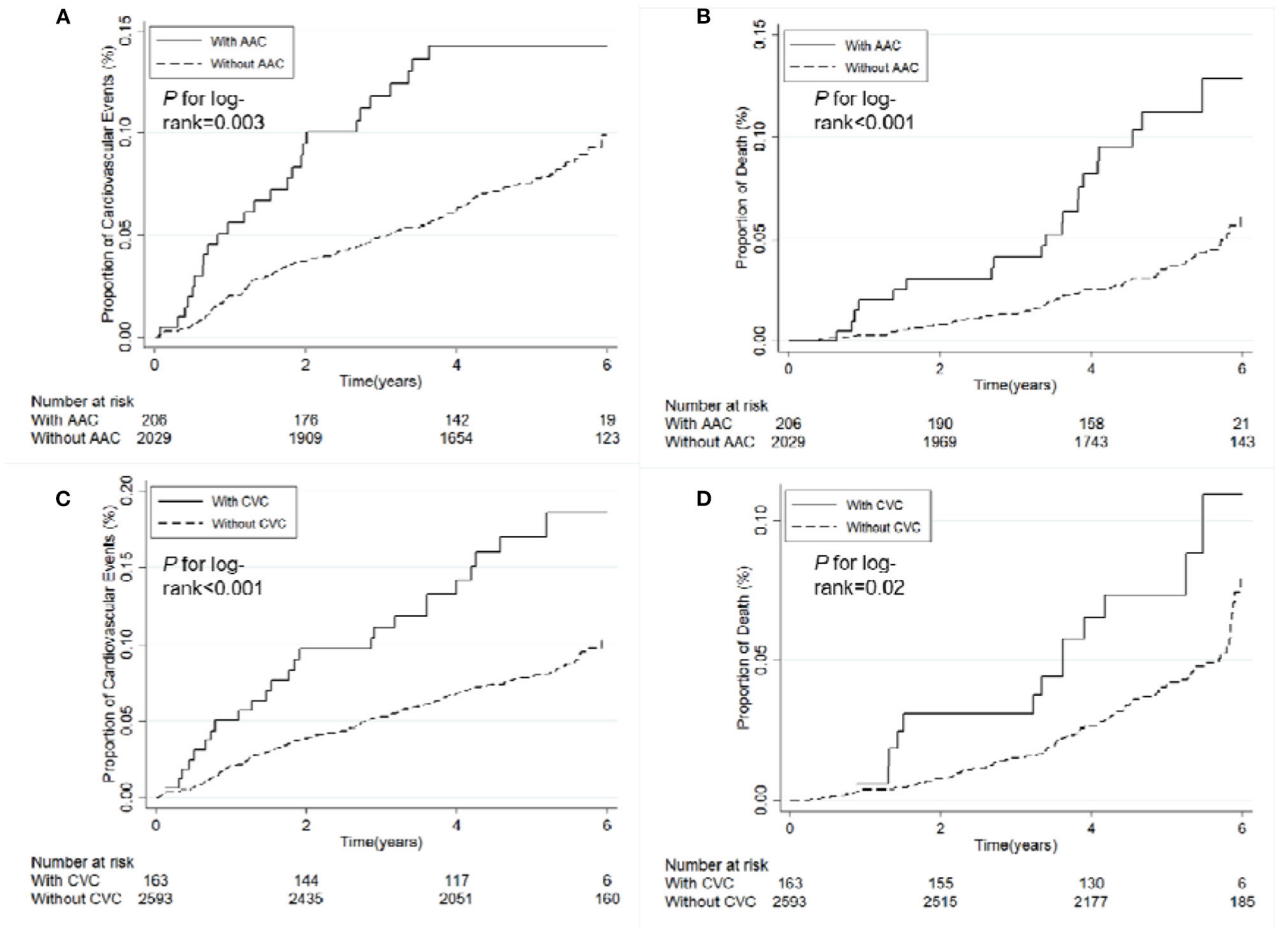
Kaplan–Meier curves for cardiovascular events or death according to presence or absence of AAC or CVC. **(A)** Category of AAC and risk of cardiovascular events. **(B)** Category of AAC and risk of death. **(C)** Category of CVC and risk of cardiovascular events. **(D)** Category of CVC and risk of death. AAC, abdominal aortic calcification; CVC, cardiac valvular calcification.

Patient survival data (Table 3, Figures 3A,B) showed that rates of cardiovascular events and all-cause mortality were significantly higher in patients with AAC than those without the disorder after a median 4.96 years (interquartile range [IQR]: 4.16–5.55 years) of follow-up for cardiovascular events and 5.05 years (IQR: 4.34–5.58 years) for all-cause mortality (log-rank *P-*values were 0.003 and <0.001, respectively). Similarly, regarding the status of CVC, cardiovascular events and all-cause mortality were significantly greater in patients with CVC compared to those without the disorder (Table 3, Figures 3C,D) after a median 4.91 years (IQR: 4.08–5.57 years) of follow-up for cardiovascular events and 5.02 (IQR: 4.22–5.63 years) for all-cause mortality (log-rank *P-*values were <0.001 and 0.02, respectively). The presence of AAC was associated with the increased risk of all-cause mortality with marginal statistical significance after the adjustment of age, gender and eGFR (HR = 1.67 [95% confidence interval [CI]:0.99–2.79]) (Table 4A). Although we did not observe any significant association between the presence of CVC and either of the events in the multivariable analysis (Table 4B), we detected a marginally significant interaction between eGFR and the CVC status for the association with cardiovascular events (*P* for interactio*n* = 0.058). The stratified analysis by the level of eGFR showed that CVC was associated with increased risk of cardiovascular events among the population with relatively normal kidney function (HR = 1.99 [95% CI:0.98–4.03] in the multivariable adjustment model among the subgroup of eGFR ≥ 45 ml/min/1.73 m^2^) rather than among those with advanced kidney disease (eGFR <45 ml/min/1.73 m^2^) (Table 5).

**Table 3:** Survival analysis for all-cause mortality and cardiovascular events with AAC or CVC as the exposure factor.

		**Patients total**	**Number of incident cases**	**Incidence rate/100 person-years**	***P* for log–rank test**
AAC	Cardiovascular events				0.003
	Yes	206	26	2.98	
	No	2,029	150	1.58	
	Total	2,235	176	1.69	
	All-Cause Mortality				<0.001
	Yes	206	20	2.14	
	No	2,029	75	0.76	
	Total	2,235	95	0.88	
CVC	Cardiovascular events				<0.001
	Yes	163	25	3.44	
	No	2,593	198	1.65	
	Total	2,756	223	1.75	
	All-Cause Mortality				0.02
	Yes	163	13	1.67	
	No	2,593	109	0.87	
	Total	2,756	122	0.92	

*AAC, abdominal aortic calcification; CVC, cardiac valve calcification*.

**Table 4:** **(A)** Cox proportional hazards regression model for all-cause mortality and cardiovascular events with AAC as the exposure factor. **(B)** Cox proportional hazard regression model for all-cause mortality and cardiovascular events with CVC as the exposure factor.

**AAC**	**Cardiovascular events**		**All-cause mortality**	
	**HR (95%CI)**	***P***	**HR (95%CI)**	***P***
**(A)**				
**Model 1**				
Yes	1.87 (1.23, 2.84)	0.003	2.84 (1.73, 4.65)	<0.001
No	Reference		Reference	
**Model 2**				
Yes	1.03 (0.67, 1.58)	0.91	1.70 (1.01, 2.86)	0.045
No	Reference		Reference	
**Model 3**				
Yes	0.99 (0.64, 1.52)	0.96	1.67 (0.99, 2.79)	0.054
No	Reference		Reference	
**CVC**	**Cardiovascular events**		**All-Cause Mortality**	
	**HR (95%CI)**	***P***	**HR (95%CI)**	***P***
**(B)**				
**Model 1**				
Yes	2.10 (1.38, 3.18)	<0.001	1.95 (1.10, 3.47)	0.02
No	Reference		Reference	
**Model 2**				
Yes	1.05 (0.68, 1.62)	0.82	1.05 (0.58, 1.91)	0.87
No	Reference		Reference	
**Model 3**				
Yes	1.06 (0.69, 1.63)	0.80	1.06 (0.58, 1.92)	0.85
No	Reference		Reference	

*Model 1 was univariate analysis, model 2 was adjusted for age and gender and model 3 was adjusted for age, gender, and eGFR.*

*CVC, cardiac valve calcification; eGFR, estimated glomerular infiltration rate; HR, hazard ratio; CI, confidence interval*.

**Table 5:** Cox proportional hazard regression model for cardiovascular events with CVC stratified by CKD stages.

**CVC**	**CKD stages 1-3a**		**CKD stages 3b-4**	
	**(eGFR≥45 ml/min/1.73m** ^**2**^ **)**		**(eGFR <45 ml/min/1.73m** ^**2**^ **)**	
	**HR (95%CI)**	***P***	**HR (95%CI)**	***P***
**Model 1**				
Yes	5.31 (2.75, 10.24)	<0.001	1.21 (0.70, 2.09)	0.50
No	Reference		Reference	
**Model 2**				
Yes	2.03 (1.00, 4.13)	0.0495	0.77 (0.44, 1.35)	0.36
No	Reference		Reference	
**Model 3**				
Yes	1.99 (0.98, 4.03)	0.06	0.78 (0.44, 1.36)	0.38
No	Reference		Reference	

*Model 1 was univariate analysis, model 2 was adjusted for age and gender and model 3 was adjusted for age, gender, and eGFR. The P-value for the age and gender adjusted association in the CKD stages 1-3a group kept three decimal places in order to show statistical significance.*

*CVC, cardiac valve calcification; CKD, chronic kidney disease; eGFR, estimated glomerular infiltration rate; HR, hazard ratio; CI, confidence interval*.

## Discussion

Cardiovascular calcification is one of the major causes for increased cardiovascular morbidity and mortality among CKD patients. In past decades, studies in CKD patients demonstrated that VC is an independent risk factor for coronary artery disease, arterial stiffness and peripheral vascular disease (14, 15). Although it was once regarded as a simply passive process of deposition of calcium and phosphate on the vascular wall, mounting experimental and clinical evidence indicates that VC is an actively regulated pathophysiological process (15, 16). Disorders in calcium-phosphorus metabolism are common in CKD patients. Long-term exposure to excessive phosphate concentrations may trigger phenotypic transformation of vascular smooth muscle cells (VSMCs) into osteoblast-like cells. This process is mediated by osteogenic transcription factors, which may lead to metastatic calcification in several sites ranging from valve tissues to vascular wall and skeleton. Traditional risk factors in cardiovascular events, such as advanced age, the presence of diabetes and hypertension, usually existing in CKD, can accelerate and aggravate the procession of VC. Cardiovascular calcification occurs subsequently. Clearly, VC isshown to be a harbinger of cardiovascular complications in patients with CKD (17).

The pathophysiology of the bone-vascular axis is complex and multifaceted, especially in CKD patients. Bone mineral disorders may infer conditions that foster VC, in turn, calcifying vessels may release several circulating factors which affect bone metabolism (18). Osteoporosis, fractures, and VC are highly prevalent in CKD patients and closely interrelated (19). This is particularly true for hemodialysis patients. In a multicenter, cross-sectional, observational study, M. Fusaro et al. demonstrated that prevalence of vertebral fractures was 55.3% in hemodialysis patients and 51.0% in the control group affected by primary osteoporosis. The prevalence of aortic VC was significantly higher in hemodialysis patients than in controls (80.6 vs. 68.4%, *P* = 0.001) (20).

Although renal transplantation can effectively corrects most of the abnormalities induced by renal insufficiency, low plasma levels of 25-hydroxyvitamin D [25(OH)D] and persistent secondary hyperparathyroidism (SHPT) are fairly common in the majority of kidney transplant patients (21, 22), which may contribute to bone mineral disorders. A high prevalence of vertebral fractures (57%) has also been reported in a renal transplant cohort (23). On the other hand, renal transplant patients are still exposed to some pro-calcifying factors (e.g., bone mineral disorders, inflammation, and immunosuppressive drugs) that undoubtedly lead to the progression of VC or *de novo* development (24).

Thus, bone lesions and VC remain the major problems for CKD patients on dialysis and even after successful renal transplantation.

Following KDIGO guidelines on chronic kidney disease—mineral and bone disorder (CKD-MBD), this current study performed lateral abdominal radiograph to evaluate AAC, and echocardiogram to detect CVC. The methods mentioned above are more widely available and less expensive than EBCT and MSCT.

In the current study, 206 (9.22%) of the 2,235 patients were found to have AAC, which was lower than the prevalence (57%) reported by another cross-sectional study from China (25).There are several potential reasons for this difference. First, patients in the earlier study had an average age of 58.18 ± 12.16, vs. 48.28 ± 13.65 in the present study. It is well-recognized that advancing age is one of the traditional risk factors for VC. Second, eGFR was higher in patients from our study because up to 71.86%of patients were from CKD stages 1 to 3, whereas in previous study all patients were from CKD 3 to 5. VC could be accelerated and aggravated with progressive decrease of renal function.

The overall AAC prevalence reported here of 9.22% in non-dialysis CKD patients at baseline is also much lower than the 72% reported in MASTERPLAN study from the Netherlands (26). The much lower prevalence of AAC observed in our study compared to the MASTERPLAN study might be attributable to the lower prevalence of diabetes and CVD (21.03 vs. 26.43%; 10.91 vs. 29.91%). Diabetes and CVD are known to be strongly associated with AAC. In addition, the number of subjects for the two studies mentioned above was quite small and included only 158 and 280 non-dialysis CKD patients, respectively.

In addition, abnormal calcium-phosphorus metabolism is often observed in CKD patients. It has been suggested that disturbances in bone and mineral metabolism contribute to the excessively high CVD morbidity and mortality among CKD patients (27). The KDIGO guidelines for CKD-MBD (6) suggest restricting the dose of calcium containing phosphate binders in the presence of arterial calcification in patients with CKD stages 3–5D and hyperphosphatemia. An open-label randomized clinical trial from Italy (28) reported that sevelamer (calcium-free phosphate binder) significantly improves cardiovascular mortality in hemodialysis patients compared to a calcium containing phosphate binder (*p* < 0.001). The IMPACT-CKD study from Thailand (29) showed that decreased active vitamin D was an independent risk for AAC in diabetes mellitus patients among CKD 3 to 5D population. This is confirmed by our finding that patients with AAC used calcium-free phosphate binder and active vitamin D preparations less often than those without. Calcium-free phosphate binder and active vitamin D preparations may play an important role in preventing the progression of AAC.

Our findings are also consistent with traditional views concerning risk factors for cardiovascular calcification. According to logistic analysis, older age, history of CVD, smoking, lower hemoglobin, hypertension, diabetes, higher LDL levels and higher serum uric acid levels increased the risk for AAC in patients with CKD Stages 1–4 (*p* < 0.05). Interestingly, female patients were more likely to have AAC. Similarly, a prospective study (30) illustrated that old women with high AAC scores had increased relative hazard for atherosclerotic vascular disease events. The mechanism underlying this relationship remains uncertain. In current study, women with AAC had an average age of 62.12 with standard deviation 8.36 years. We hypothesize that is in part due to the increase in bone demineralization, especially in postmenopausal women.

The Framingham study demonstrated that AAC is a predictor for cardiovascular morbidity and mortality in the general population (31, 32). Subsequently, a single center cohort study from Japan reported a significant association between the presence of AAC and both all-cause and cardiovascular mortality in hemodialysis patients (12). Recently, a subgroup analysis in the MASTERPLAN study (26) illustrated that screening for AAC can identify non-dialysis patients at high risk for cardiovascular events. Findings of the current study echoed these views.

In our study, 2,235 patients with CKD stages 1–4 were included. The rates of cardiovascular events and all-cause mortality were significantly higher in patients with AAC than those without the disorder after a median 4.96 years of follow-up for cardiovascular events and 5.05 years for all-cause mortality. Higher adverse outcomes incidence was observed in enrolled patients with AAC. After adjustment for age, sex and eGFR, AAC was associated with increased risk of all-cause mortality.

CVC is also a common finding in dialysis patients and has been associated with the severity of cardiovascular diseases (15, 33). A prospective cohort study (34) involving 144 adult CKD-5D patients reported that the incidence of mitral valve calcification (MVC) was 38.2% and aortic valve calcification (AVC) was 44.4%. Patients with the combined presence of AVC and MVC had a 2-fold increased risk of all-cause mortality compared to patients without CVC.

Despite these findings, the association between CVC and the outcomes of non-dialysis patients is still illusive. A recent cross-sectional study in South Korea (13) demonstrated that pre-dialysis CKD patients with CVC had more severe coronary artery disease than those without any valve calcification. In addition, in that study the prevalence of CVC in pre-dialysis group (eGFR <60 ml/min/1.73 m^2^) was higher than that in non-CKD group (eGFR≥60 ml/min/1.73 m^2^) (18.1 vs. 4.5%, *p* < 0.001), which was consistent with our findings. In current study, the prevalence of CVC increased with the progressive decrease of renal function. However, the prevalence of 5.91% CVC among 2,756 non-dialysis patients with 1–4 stages was considered to be lower than that reported in a Korean population. Another retrospective study reported a prevalence of 15.28% CVC in 288 Chinese inpatients with CKD (28). These discrepancies in prevalence are likely attributable to differences in the study population age, eGFR, history of CVD and DM.

This study also found that patients with CVC had inadequate nutrition, and levels of Hb, Alb, cholesterol and LDL-C were significantly lower in patients with CVC. In the general population, hypercholesterolemia is a conventional risk factor for CVD. Interestingly, in the present study cholesterol and LDL-C levels were significantly lower in patients with than without CVC. This was also reported by previous studies (35, 36). The mechanism underlying this relationship remains uncertain. This phenomenon could be explained as follows: First, malnutrition and chronic inflammation is common in CKD patients, which could reduce the level of cholesterol (37). Second, patients with CVC were more likely to have CVD, DM (31.29 vs. 10.35%, *p* < 0.001; 39.26 vs. 20.29%, *p* < 0.001, respectively) and higher BMI (25.03 ± 3.49, 24.34 ± 3.61, *P* = 0.019)compared with those without CVC. Our previous study reported that the proportion of statin treatment was 37.9% in the CVD patients vs. 17.0% in the non-CVD patients (3). Therefore, patients with CVC might use hypolipidemic drugs more frequently and earlier before enrolled in our study.

We observed that cardiovascular events and all-cause mortality were significantly greater in patients with CVC compared to those without the disorder after a median 4.91 years of follow-up for cardiovascular events and 5.02 years for all-cause mortality cardiovascular events and all-cause mortality were significantly greater in patients with CVC. After adjustment for age, sex, and eGFR, CVC was associated with increased risk of cardiovascular events only in patients with CKD stages 1 to 3a.

Our study had several limitations. In clinical practice, quantitative evaluation of AAC or CVC might present variability due to different observers from 39 clinical centers located at 28 cities in 22 provinces of China. Therefore, the present study didn't quantitatively assess the calcification levels of AAC and CVC. The relationship between severity of AAC or CVC and outcomes of non-dialysis remains unclear. Further research is required to evaluate the calcification levels.

In conclusion, demographic and traditional cardiovascular risk factors were associated with cardiovascular calcification, especially AAC. We observed that incidence of cardiovascular events and all-cause mortality were significantly greater in enrolled patients with AAC or with CVC during the follow-up. AAC may be associated with risk of death for patients CKD of any severity, while CVC as a possible risk factor for cardiovascular disease only among those with mild to moderate CKD. Clinicians should be aware of the importance of screening for AAC or CVC in CKD patients, especially in the early stage of CKD. Further studies among pre-dialysis CKD patients, especially those with long follow-up time, were warranted to examine the associations.

## Data Availability Statement

The original contributions presented in the study are included in the article/Supplementary Material, further inquiries can be directed to the corresponding author/s.

## Ethics Statement

The studies involving human participants were reviewed and approved by Ethics Committee of Peking University First Hospital. The patients/participants provided their written informed consent to participate in this study.

## Author Contributions

C-STRIDE study data collection was the result of the joint efforts of 39 centers of the collaborative group. M-HZ and LXZ are the main initiators and designers of the C-STRIDE study. We acknowledge KH for the efforts in study design. XQW is the head of the subcenter. In this study, statistical analyses were conducted by JWW and LW. HC, XRZ, JY, WJW, and SPH were mainly responsible for subjects recruitment and data entry. LW and XQW were responsible for paper writing. All authors contributed to the article and approved the submitted version.

## Funding

This study was supported by grants from the National Natural Science Foundation of China (81874439, 91846101, 81771938, 81301296, and 81900665), the National Natural Science Foundation for Young Scholars of China (81703991), Scientific Research Project for Department of Education of Hubei Province (D20202003), Beijing Nova Programme Interdisciplinary Cooperation Project (Z191100001119008), the National Key R&D Program of the Ministry of Science and Technology of China (2016YFC1305405, 2019YFC2005000), Chinese Academy of Medical Sciences Research Unit (No. 2019RU023), CAMS Innovation Fund for Medical Sciences (2019-I2M-5-046), the University of Michigan Health System-Peking University Health Science Center Joint Institute for Translational and Clinical Research (BMU20160466, BMU2018JI012, and BMU2019JI005), PKU-Baidu Fund (2019BD017), and from Peking University (BMU2018MX020, PKU2017LCX05).

## The Chinese Cohort Study of Chronic Kidney Disease (C-STRIDE) Collaborators

Ming-Hui Zhao, Luxia Zhang, Peking University First Hospital; Xiaoqin Wang, Jun Yuan, the Affiliated Hospital of Hubei Traditional Chinese Medical College; Qiaoling Zhou, Qiongjing Yuan, the Xiangya Hospital of Central South University; Menghua Chen, Xiaoling Zhou, General Hospital of Ningxia Medical University; Shuxia Fu, Shaomei Li, the Second Hospital of Hebei Medical University; Yan Zha, Rongsai Huang, Guizhou Provincial People's Hospital; Zhangsuo Liu, JunJun Zhang, the First Affiliated Hospital of Zhengzhou University; Li Wang, Lei Pu, Sichuan Academy of Medical Sciences and Sichuan Provincial People's Hospital; Jian Liu, Suhua Li, the First Affiliated Hospital of Xinjiang University of Medicine; Zuying Xiong, Wei Liang, Peking University Shenzhen Hospital; Jinghong Zhao, Jiao Mu, Xinqiao Hospital; Xiyan Lian, Yunjuan Liao, the Second Affiliated Hospital of Kunming Medical College; Hua Gan, Liping Liao, the First Affiliated Hospital of Chongqing University of Medicine; Rong Wang, Zhimei Lv, Shandong Provincial Hospital; Yunhua Liao, Ling Pan, the First Affiliated Hospital of Guangxi University of Medicine; Xiaoping Yang, Zhifeng Lin, the First Affiliated Hospital of the Medical College, Shihezi University; Zongwu Tong, Yun Zhu, Yuxi City People's Hospital; Qiang He, Fuquan Wu, Beilun People's Hospital in Ningbo; Rong Li, Kai Rong, the Second Affiliated Hospital of Tianjin University of Medicine; Caili Wang, Yanhui Zhang, the First Affiliated Hospital of Baotou Medical College; Yue Wang, Wen Tang, Peking University Third Hospital; Hua Wu, Ban Zhao, Beijing Hospital of Ministry of Health; Rongshan Li, Lihua Wang, the Second Hospital of Shanxi University of Medicine; Detian Li, Feng Du, Shengjing Hospital of China Medical University; Yonggui Wu, Wei Zhang, the First Affiliated Hospital of Anhui University of Medicine; Shan Lin, Pengcheng Xu, Tianjin Medical University General Hospital; Hongli Lin, the First Affiliated Hospital of Dalian University of Medicine; Zhao Hu, Fei Pei, Shandong University Qilu Hospital; Haisong Zhang, Yan Gao, the Affiliated Hospital of Hebei University; Luying Sun, Xia Li, Dongzhimen Hospital Affiliated to Beijing University of Chinese Medicine; Wenke Wang, Fengling Lv, Chifeng Second Hospital; Deguang Wang, Xuerong Wang, the Second Affiliated Hospital of Anhui University of Medicine; Dongmei Xu, Lijun Tang, Qianfoshan Hospital; Yingchun Ma, Tingting Wang, China Rehabilitation Research Center, Beijing Boai Hospital; Ping Fu, Tingli Wang, West China Hospital of Sichuan University; Changying Xing, Chengning Zhang, the First Affiliated Hospital with Nanjing Medical University; Xudong Xu, Haidong He, Minhang Central Hospital; Xiaohui Liao, Shuqin Xie, the Second Affiliated Hospital of Chongqing University of Medicine; Guicai Hu, Lan Huang, the Affiliated Hospital of Chengde Medical College.

## Conflict of Interest

The authors declare that the research was conducted in the absence of any commercial or financial relationships that could be construed as a potential conflict of interest.

## Publisher's Note

All claims expressed in this article are solely those of the authors and do not necessarily represent those of their affiliated organizations, or those of the publisher, the editors and the reviewers. Any product that may be evaluated in this article, or claim that may be made by its manufacturer, is not guaranteed or endorsed by the publisher.
