# Supplementary material for: Prevalence and Correlates of Cardiovascular Calcification and Its Prognostic Effects Among Patients With Chronic Kidney Disease: Results From the C-STRIDE Study
Source: Front Public Health. 2022 Jan 6;9:762370. doi: 10.3389/fpubh.2021.762370 (PMC8771912; doi:10.3389/fpubh.2021.762370)
Supplement: Supplementary file 1 [file Data_Sheet_1.PDF]

Supplemental table 1. Univariate logistic regression analysis for AAC in patients with CKD Stages 1 to 4

| Characteristic | OR | 95%CI | *P* |
|---|---|---|---|
| Age, per 1 year increase | 1.10 | 1.08-1.11 | <0.001 |
| Gender, male vs. female | 0.89 | 0.67-1.19 | 0.44 |
| BMI, per 1 kg/m$^2$ | 1.04 | 1.00-1.09 | 0.04 |
| History of CVD, yes vs. no | 3.66 | 2.6-5.15 | <0.001 |
| Tobacco use, yes vs. no | 1.43 | 1.06-1.91 | 0.02 |
| Hemoglobin, per 1 g/L increase | 0.98 | 0.98-0.99 | <0.001 |
| Serum albumin, per 1 g/L increase | 1.00 | 0.98-1.02 | 0.80 |
| 24-h urine protein, per 1 natural log increase (g/24h) | 1.06 | 0.95-1.19 | 0.27 |
| Hypertension, yes vs. no | 3.44 | 2.04-5.80 | 0.001 |
| Diabetes, yes vs. no | 4.14 | 3.08-5.56 | <0.001 |
| Triglyceride, per 1 natural log increase (mmol/L) | 1.01 | 0.77-1.31 | 0.97 |
| Cholesterol, per 1 natural log increase (mmol/L) | 0.87 | 0.60-1.27 | 0.48 |
| LDL, per 1 natural log increase (mmol/L) | 1.22 | 0.83-1.79 | 0.31 |
| eGFR, per 1 ml/min/1.73m$^2$ increase | 0.99 | 0.98-0.99 | <0.001 |
| Serum uric acid, per 1 umol/L increase | 1.002 | 1.001-1.003 | 0.001 |
| Serum calcium, per 1 mmol/L increase | 1.06 | 0.53-2.14 | 0.87 |
| Serum phosphorus, per 1 natural log increase (mmol/L) | 1.12 | 0.58-2.15 | 0.74 |
| IPTH, per 1 natural log increase (pg/ml) | 1.41 | 1.14-1.74 | 0.001 |
| Calcium-free phosphorus binder, yes vs. no | 0.58 | 0.37-0.92 | 0.02 |
| Active vitamin D, yes vs. no | 0.57 | 0.33-0.96 | 0.03 |

Supplemental table 2. Univariate logistic regression analysis for CVC in patients with CKD Stages 1 to 4

| Characteristic | OR | 95%CI | *P* |
|---|---|---|---|
| Age, per 1 year increase | 1.12 | 1.10-1.15 | <0.001 |
| Gender, male vs. female | 0.94 | 0.68-1.29 | 0.69 |
| BMI, per 1 $kg/m^2$ | 1.06 | 1.01-1.10 | 0.02 |
| History of CVD, yes vs. no | 3.63 | 2.55-5.17 | <0.001 |
| Tobacco use, yes vs. no | 1.08 | 0.78-1.51 | 0.64 |
| Hemoglobin, per 1 g/L increase | 0.99 | 0.98-0.99 | <0.001 |
| Serum albumin, per 1 g/L increase | 0.98 | 0.96-1.00 | 0.03 |
| 24-h urine protein, per 1 natural log increase (g/24h) | 0.91 | 0.80-1.02 | 0.10 |
| Hypertension, yes vs. no | 1.53 | 0.98-2.37 | 0.06 |
| Diabetes, yes vs. no | 2.54 | 1.83-3.53 | <0.001 |
| Triglyceride, per 1 natural log increase (mmol/L) | 1.03 | 0.77-1.38 | 0.86 |
| Cholesterol, per 1 natural log increase (mmol/L) | 0.61 | 0.39-0.96 | 0.03 |
| LDL, per 1 natural log increase (mmol/L) | 0.45 | 0.30-0.67 | <0.001 |
| eGFR, per 1 $ml/min/1.73m^2$ increase | 0.99 | 0.98-0.99 | <0.001 |
| Serum uric acid, per 1 umol/L increase | 1.00 | 0.998-1.001 | 0.79 |
| Serum calcium, per 1 mmol/L increase | 0.75 | 0.34-1.63 | 0.47 |
| Serum phosphorus, per 1 natural log increase (mmol/L) | 0.69 | 0.32-1.48 | 0.34 |
| IPTH, per 1 natural log increase (pg/ml) | 1.13 | 0.89-1.43 | 0.32 |
| Calcium-free phosphorus binder, yes vs. no | 1.20 | 0.81-1.79 | 0.36 |
| Active vitamin D, yes vs. no | 1.46 | 0.98-2.18 | 0.07 |
